# Effect of the Combination of Ultra-High-Molecular-Weight Polyethylene, Denim Fabric, and Aluminum on the Functional Properties in Composite Crash Boxes

**DOI:** 10.3390/polym18141709

**Published:** 2026-07-12

**Authors:** Baran Erkek, Mehmet Şükrü Adin, Ertan Kosedag, Mateusz Bronis, Ayşe Didem Erol Erkek, Hamit Adin

**Affiliations:** 1 Van Vocational School, Van Yuzuncu Yil University, Van 65080, Turkey; 2 Besiri OSB Vocational School, Batman University, Batman 72060, Turkey; 3 Faculty of Engineering, Van Yuzuncu Yil University, Van 65080, Turkey; 4 Department of Machine Design and Machining, Kielce University of Technology, 25314 Kielce, Poland; mateuszbronisck@gmail.com; 5 Faculty of Engineering and Architecture, Batman University, Batman 72100, Turkey; hamit.adin@batman.edu.tr

**Keywords:** crash boxes, combination, denim, polymer composites, ultra-high-molecular-weight polyethylene

## Abstract

Vehicle crash boxes are elements that protect the integrity of vehicles and ensure the safety of occupants in potential vehicle accidents. These crash boxes are mounted on the chassis of vehicles. In this study, composite crash boxes fabricated from aluminum, which is known for its lightweight properties, as well as denim and ultra-high-molecular-weight polyethylene, both of which are widely available on the market, were investigated experimentally. Composite crash boxes composed of an epoxy resin matrix reinforced with denim fabric (DenimFRP) and ultra-high-molecular-weight polyethylene (UHMWPEFRP) fibers, as well as aluminum (Al), were produced. The crash boxes were manufactured using a vacuum infusion method. This combination was produced by wrapping these fibers around an aluminum core. The energy absorption values, peak force values, and specific energy absorption values of the manufactured crash boxes were obtained through quasi-static compression tests and then compared. The best energy absorption value was achieved with the Al+denimFRP composite crash box manufactured by wrapping denim around aluminum at a workload of 1645.22 J, and its specific energy absorption value was also calculated as 15.52 J/g. The difference between the highest and lowest energy absorption was determined to be 244.61%. The highest peak strength value was obtained with the Al+denim+UHMWPEFRP sample, which contained a combination of aluminum on the inside, denim fabric in the middle, and UHMWPE fabric on the outside. Among the individually produced samples, the Al+denimFRP composite crash box manufactured with denim fiber exhibited higher results compared with the UHMWPEFRP composite box manufactured with ultra-high-molecular-weight polyethylene.

## 1. Introduction

Vehicle crash boxes are among the most important safety elements of vehicles. The purpose of these crash boxes is to protect the vehicle’s integrity in the event of a potential accident. While preserving the vehicle’s integrity, they must have good energy absorption performance. The primary function of a crash box is not to remain rigid, but rather to absorb impact energy and minimize the amount of energy transmitted to the rest of the vehicle. Vehicle crash boxes produced for this purpose are mounted on the chassis at the front of vehicles. Many studies have been carried out with different materials used to manufacture these crash boxes, which are generally made of metallic lightweight materials. Composite materials are also among these. Among composite materials, studies on carbon and glass-fiber-reinforced composite materials, which are generally the most common on the market, are prominent [1,2,3,4,5,6]. The fiber type in the composites plays a significant role in the mechanical properties of such materials, and its interface with the matrix is effective. In addition to the fiber type, researchers sometimes try to improve the properties of composite materials by combining several materials [7,8,9,10].

Researchers have attempted to improve energy absorption by modifying the geometry of composite crash boxes [11,12]. Özen et al. [11] conducted research on composite crash boxes featuring hollow and cellular fillings. Using carbon fiber, the researchers produced square, hexagonal, and cylindrical crash boxes, the shapes of which were square and hexagonal cell forms. At the end of their study, they concluded that cellular filling was the most suitable crash box design. Bunsri et al. [13] compared the energy absorption properties of crash boxes with cellular structures. They stated that crash boxes that had filled structures provided better results than traditional crash boxes. Hussain et al. [14] manufactured crash boxes with four different cross-sections (square, cylindrical, hexagonal, and decagonal). They subjected glass fiber samples to impact tests. They stated that they obtained the best results in trigger-free crash boxes with decagonal crash boxes. Awd Allah et al. [15] investigated the cellular configuration and polyurethane foam filling of square glass-fiber-reinforced composite tubes. They subjected the foam core to a quasi-static lateral compression test to investigate the effects of single- and multi-cell foam fillings on them. They concluded that the square structure with single-cell foam filling yielded the best results. The effects of geometry on crash boxes have been reported in previous studies. Furthermore, the additives incorporated into them or the combination processes are attracting the attention of researchers [16,17,18,19,20]. Abd El Alal et al. [21] conducted research on crash-resistant composites based on the combination of glass and carbon fibers. In their study, they emphasized the importance of the order of combinations and suggested their benefits as energy absorbers in automotive applications. Lu et al. [22] experimentally investigated the crashing behavior of carbon/glass composite pipes. They determined that the energy absorption capacities of the composites could be increased by optimizing fiber volume fraction and the number of layers. In another study, Awd Allah et al. [23] investigated the behavior of hybrid glass/nano-silica/epoxy composite cylinders under lateral loading. They concluded that the addition of SiO_2_ to the combined glass/nano-silica/epoxy composites reduced the specific energy absorption of the composites. It was emphasized in many studies that particles such as nanoparticles, natural clays or metal powders added to composites have an effect on mechanical properties [6,24,25,26,27]. In addition to fiber-to-fiber combination, the integration of metal to fiber components has become a focal point for researchers seeking to optimize structural performance [18]. El-Baky et al. [28] investigated the crashworthiness of aluminum (Al) cylinders wrapped with jute (J)/glass (G)-reinforced epoxy composite. They concluded that a crash box with the combination order of Al-3G-2J-3G would be suitable to use in automobiles. Zhang et al. [29] investigated the fracture performance and energy absorption properties of aluminum/CFRP combination thin-walled tubes. They emphasized that increasing the number of layers had little effect on the failure mode of the tubes but significantly affected energy absorption. Hwang et al. [30] used two types of aluminum-composite tubes and one type of pure braided composite tube to investigate the crashworthiness of composite tubes. They concluded that the specific energy absorption of braided tubes is always higher than that of prepreg tubes. Normal polyethylene molecules are not oriented and are easily separated from each other. Studies have been conducted to make these fibers stronger. To improve the strength of the fibers, it is necessary to increase the orientation along the fiber axis and the proportion of crystalline regions. In order to carry out these processes, long molecular chains are required; therefore, ultra-high-molecular-weight polyethylene (UHMWPE) is used as the raw material for high-performance polyethylene fibers [31]. UHMWPE has gained a place in the production of composite materials due to its low-density, high-strength fiber structure [32,33]. Khatiwada et al. [34] conducted research on nanocomposites consisting of ultra-high-molecular-weight fabrics containing single-walled nanotubes. They reported that nanocomposites and pure composites yielded better results than aluminum sheets. Jian et al. [35] investigated the effect of CNT grafting on the mechanical properties of UHMWPE fiber/HDPE composites. They reported that a 4% CNT ratio yielded the best performance.

Denim fabrics can be produced with a wide variety of properties. The most distinctive feature of these fabrics is that warp yarns are dyed indigo and weft yarns are in their natural color (white). Classic denim weaves are 3/1 Z twill, where warp yarns dominate the fabric surface, while weft yarns dominate the reverse. Therefore, the front appears blue and the reverse white. The warp density varies between 24 and 27 picks/cm, while the weft density can vary between 15 and 18 picks/cm. Furthermore, according to the TS 2791 standard, the weight of denim fabrics varies between 271 and 466 g/m^2^. Their tensile strength varies between 356 and 801 N in the warp direction and 178 and 312 N in the weft direction [36]. Studies on composite materials conducted using denim fabric can be found in the literature. Denim composites have applications such as sound-absorbing materials, composites made from recycled denim, fire-retardant composites, impact-absorbing composites, and biocomposites [37,38,39,40,41].

In the literature, numerous studies conducted by researchers on crash boxes and composite pipes can be encountered. These studies have generally focused on the combination of commonly used fiber types such as glass fiber, carbon fiber, and other types of fibers. Metal–fiber combinations were also employed in some of the studies. In our study, crash boxes were produced using aluminum (Al), ultra-high-molecular-weight polyethylene (UHMWPE) fabric, and denim fabric, which were also combined. Ultra-high-molecular-weight polyethylene (UHMWPE) fabric was chosen as the fiber for composite crash boxes due to its high durability, while denim fabric was chosen due to its widespread availability. Vacuum infusion was chosen as the production method. This method prevented air pockets in the composite and ensured a more homogeneous mixture. All produced samples were subjected to quasi-static compression testing. After the test, the energy absorption values, specific energy absorption values, and peak force values of the samples were compared using the obtained data. Following the quasi-static compression tests, samples were examined under a digital microscope for damage analysis.

## 2. Materials and Methods

### 2.1. Materials and Properties

Composite crash boxes were produced using epoxy resin matrices as single (aluminum, denim, and UHMWPE) and combined (Al+UHMWPE, Al+denim, and Al+denim+UHMWPE) materials. Hegzon LR160 and Hegzon LH160, supplied by Dost Kimya Limited (Istanbul, Turkey), were used as epoxy resin and hardener. The hardener and epoxy resin were mixed in a 1/4 ratio. The properties of the epoxy resin are given in Table 1. The density of UHMWPE fabric was 294 g/m^2^, and the density of denim fabric was 267 g/m^2^. UHMWPE fabric has a weft density of 16 picks/cm and a warp density of 25 picks/cm. Denim fabric has a weft density of 23 picks/cm and a warp density of 25 picks/cm. Both fabrics were 3/1 Z twill weave. Samples, excluding aluminum, were initially produced individually. The single windings were wrapped in nine layers and then wrapped evenly around the aluminum to achieve a 100 mm size. During this combination, denim fabric was first wrapped around the aluminum (Al+denimFRP), with the aluminum facing inward. UHMWPE fabric was then wrapped around the aluminum (Al+UHMWPEFRP), and finally, denim and UHMWPE (Al+denim+UHMWPEFRP) were wrapped around the aluminum, respectively, to achieve the combination. If only denim or UHMWPE was to be wrapped around the aluminum, the wrapping was done with six layers. If both denim and UHMWPE were to be wrapped around the aluminum, the wrapping was done with three layers each of denim and UHMWPE.

### 2.2. Preparation of Composites

Air tightness is crucial for the vacuum infusion method. A leak-proof surface was achieved by wrapping tape around the cardboard coils. This prevented the epoxy resin from coming into contact with the cardboard bobbin. After this process, denim and UHMWPE were wrapped around the cardboard bobbins. For the combined samples, six layers of denim fabric were wrapped around the aluminum for Al+denimFRP, and six layers of UHMWPE fabric were wrapped around it for Al+UHMWPEFRP. Specific properties of the samples are listed in Table 2.

**Figure 1:**
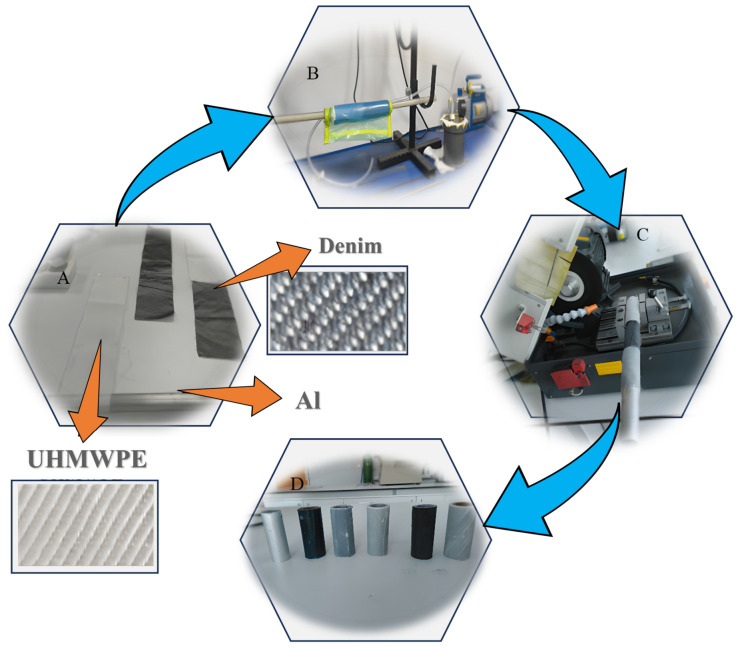
The production phase of composite crash boxes is as follows: (**A**) winding the fabrics onto the bobbin and aluminum tube, (**B**) applying the epoxy resin by vacuum infusion, (**C**) bringing the samples to the desired size, and (**D**) end products.

Finally, for Al+denim+UHMWPEFRP, three layers of denim and three layers of UHMWPE fabric were wrapped around the aluminum. Separator fabric and a grid were then wrapped, and the final vacuuming process was completed using nylon to ensure sealing. Following this process, epoxy resin was applied on one side and vacuumed on the other. The samples were allowed to stay at room temperature for one day. All samples were then cut to 100 mm for testing using a Metkon Matecut 250 device. The production process of the crash boxes and the final shapes are shown in Figure 1. The denim and UHMWPE composite crash boxes, wrapped around cardboard cores, were then soaked in water to allow the cardboard core to dissolve before testing.

### 2.3. Procedures for Crash Testing

**Figure 2:**
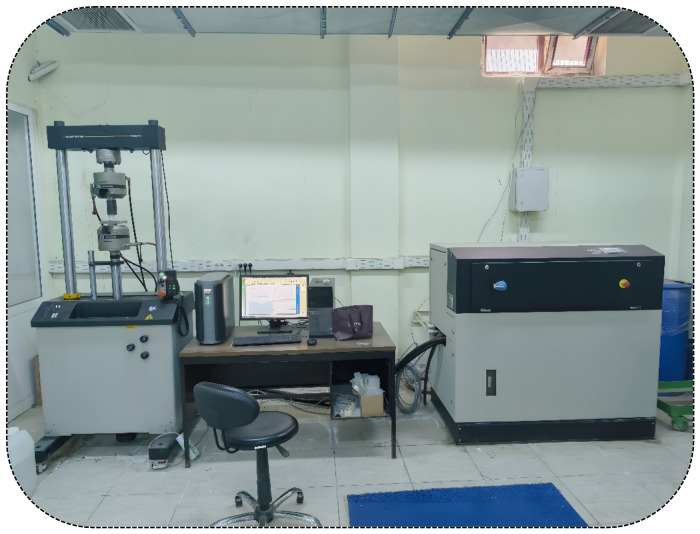
Experimental setup for the quasi-static axial compression test.

After the production phase, the composite crash boxes were subjected to quasi-static compression tests to obtain the necessary experimental data. Although these loading conditions do not represent actual crashing conditions, they were used in the calculations of peak force energy absorption and specific energy absorption [10,12]. Tests were conducted at room temperature using a 100 kN Instron BS 8801 device (Figure 2). The test speed was selected as 2 mm/min. Load-displacement curves were plotted using the data obtained from the test, which compressed the samples to 30 mm. Energy absorption values for the samples were calculated from this curve. MATLAB (Matlab R 2020a)was used for the calculation. The area under the curve for the energy absorption value was calculated. Peak force values for the samples were also another output obtained from the test. Finally, the specific energy absorption value, used to compare different samples, was obtained by dividing the energy absorption values by the sample weights.

## 3. Results and Discussion

In this section, the load-displacement curves obtained after testing for all samples are evaluated. Using these curves, peak force, energy absorption values, and specific energy absorption values are calculated and compared.

**Figure 3:**
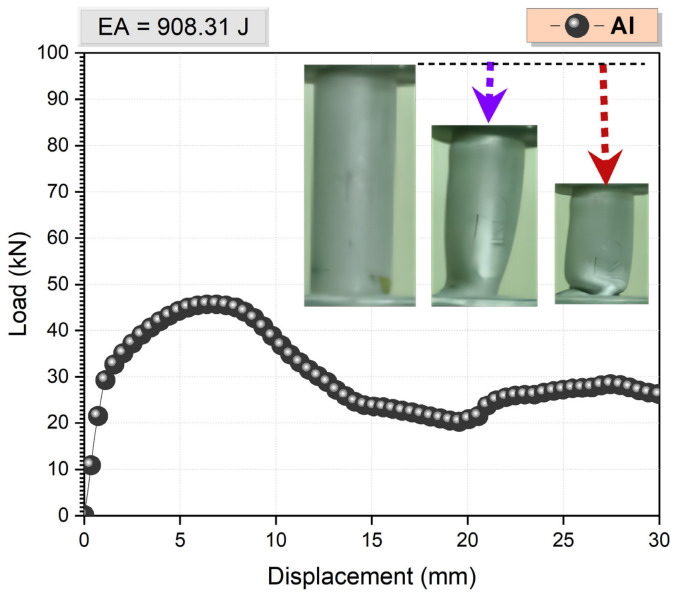
Length change and load-displacement graph of the aluminum (Al) sample during the test.

The maximum peak force obtained from the Al sample was 45.83 kN. During the test, a buildup of material was observed, starting at the bottom of the sample (Figure 3). Calculating the area under the load-displacement curve yielded the sample’s energy absorption value. The calculated energy absorption value was 908.31 J. This value was the highest among all individual samples, surpassing those of the other denim and UHMWPE samples. The specific energy absorption value, another calculated performance metric, was used to compare different sample types. Dividing the energy absorption value by the sample’s weight of 65.34 g yielded the value of 13.90 J/g.

**Figure 4:**
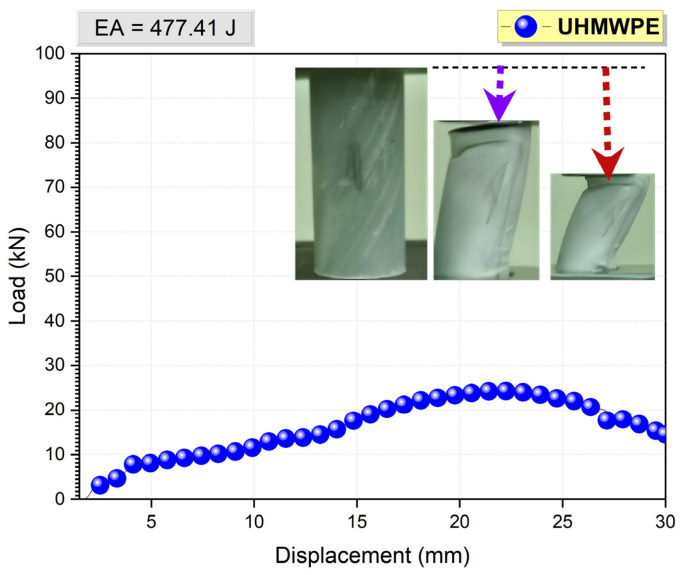
Length change and load-displacement graph of the UHMWPEFRP sample during the test.

The other sample was the UHMWPEFRP composite crash box with an ultra-high- molecular-weight polyethylene fiber matrix. The peak force obtained for this sample was 24.32 kN. This value is almost half that of the aluminum crash box, which is metal. The load-displacement curve for this sample is shown in Figure 4. Calculating the area under the load-displacement curve, the energy absorption value was found as 477.41 J. This value, like the peak force value, is almost half that of the Al sample. The specific energy absorption value of the UHMWPEFRP, which weighs 73.10 g, was calculated as 6.53 J/g. Unlike the other two values, this value is less than half that of the Al sample. The Al sample’s metallic nature can be explained by its superior mechanical properties. No delamination was observed in the sample during the test. The sample tended to bend rather than undergo crashing (Figure 4). The matrix and fibers maintained their integrity without separating from each other.

**Figure 5:**
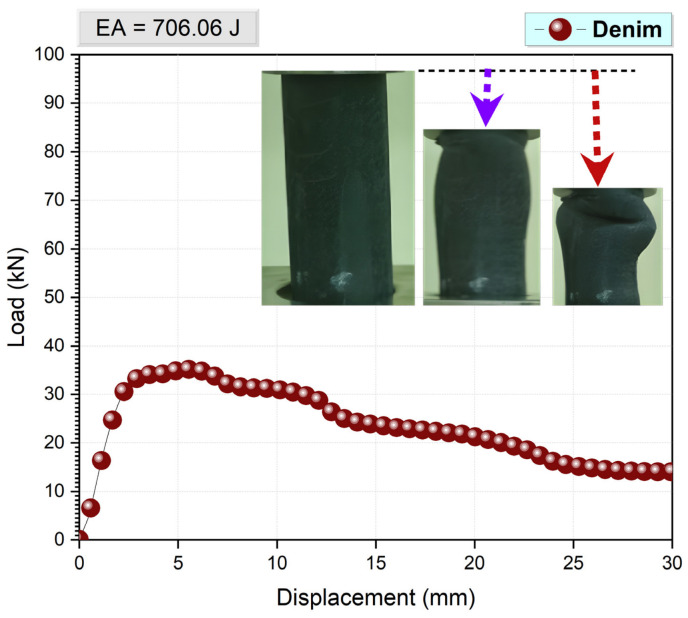
Length change and load-displacement graph of the DenimFRP sample during the test.

The DenimFRP sample failed to meet the values of the Al sample but showed better results than the UHMWPEFRP sample. In this sample, a commercially available denim fabric fiber was used. The peak force value obtained was 35.15 kN. This value is approximately halfway between the Al sample and the UHMWPEFRP sample. The energy absorption value was calculated as 706.06 J using the area under the load-displacement curve (Figure 5). This value is 202.25 J less than the Al sample, but 228.65 J more than the UHMWPEFRP sample. Because the sample weight was 65.09 g, the calculated specific energy absorption value was 10.84 J/g, which is 3.06 J/g less than the Al sample and 4.31 J/g more than the UHMWPEFRP sample. This sample also exhibited similar behavior to the UHMWPEFRP sample during the test. Although no significant deformation was observed, the sample tended to bend under the impact load. After the experiment, it exhibited a tendency to recover to its original shape but was unable to do so.

**Figure 6:**
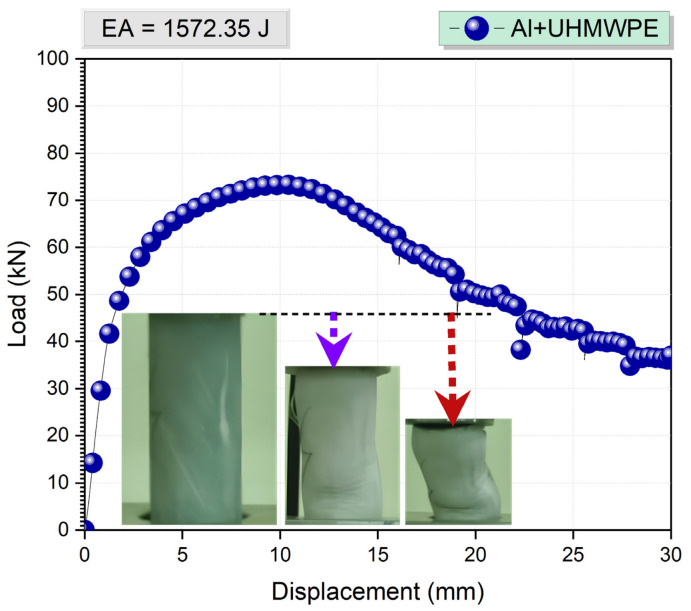
Length change and load-displacement graph of the Al+UHHMWPEFRP sample during the test.

The Al+UHMWPEFRP sample, one of our combined samples, exhibited results similar to the mechanical properties of the individual Al and UHMWPEFRP samples. The peak force value was calculated as 73.38 kN. This value is 3.23 kN higher than the total peak force values of the individual Al and UHMWPEFRP samples. A similar trend was observed for the energy absorption value. The energy absorption value calculated for this sample was 1572.35 J. This value is 186.63 J higher than the total energy absorption values of the combined Al and UHMWPEFRP samples. These values are also higher than those of the DenimFRP samples. This sample weighed 111.79 g, and the specific energy absorption was calculated as 14.06 J/g. This value, as with the peak force and energy absorption values, is no more than the total specific energy values of the individual Al and UHMWPEFRP samples. The length change and load-displacement graph of this sample during the test are given in Figure 6. No delamination was observed during the test (Figure 6). The matrix and fibers did not separate from each other. The aluminum sample wrapped with the UHMWPE tended to assume a similar shape to that observed when it was subjected to a quasi-static test alone. However, the outer UHMWPE wrap partially restrained this deformation. Subsequently, the UHMWPE layer began to fold.

**Figure 7:**
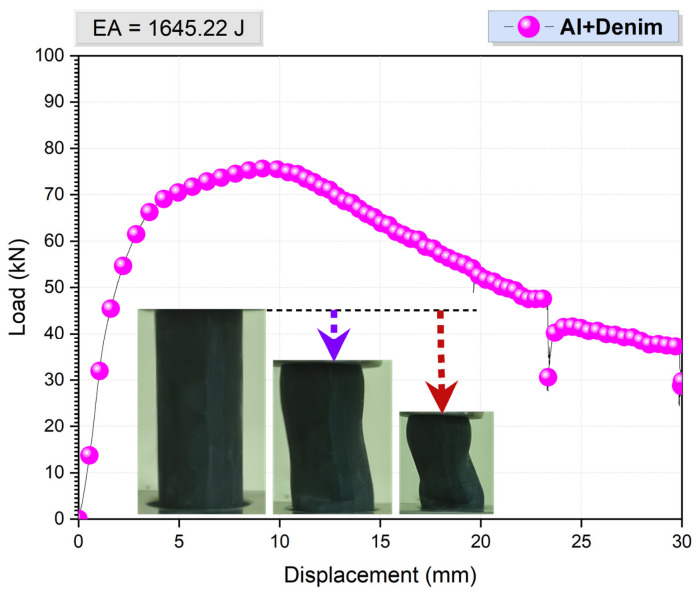
Length change and load-displacement graph of the Al+denimFRP sample during the test.

Another combined sample was the Al+denimFRP crash box, which had an aluminum interior and denim wrapped around it. The peak force obtained with this sample was 75.74 kN. This value is 5.24 kN lower than the total peak force values obtained separately from the Al and DenimFRP samples. When the energy absorption value was calculated, the value was found as 1645.22 J. Comparing this value with the total energy absorption values of the individual Al and DenimFRP samples, it was established that there was only a slight difference; it is 30.85 J more than the total value of Al and DenimFRP samples. Dividing the energy absorption value by the sample weight of 105.99 g, the specific energy absorption value of 15.52 J/g was obtained. This value is 9.22 J/g less than the total specific energy absorption values of the individual Al and DenimFRP samples. The denimFRP sample exhibited higher values than the UHMWPEFRP sample. During the test, the Al+denimFRP sample exhibited higher energy absorption and specific energy absorption values of 72.87 J and 1.46 J/g, respectively, compared with the Al+UHMWPEFRP sample. The load-displacement graph and the length change during the test for this sample are given in Figure 7. No deformation due to fiber breakage or delamination was observed during the test (Figure 7). It exhibited a crashing behavior similar to that of the other combined sample, Al+UHMWPEFRP. The aluminum on the inside the sample tended to fold, but the denim on the outside tended to suppress it to some extent. The conditions resulting from those folds are observed in the load-displacement graph (Figure 7).

**Figure 8:**
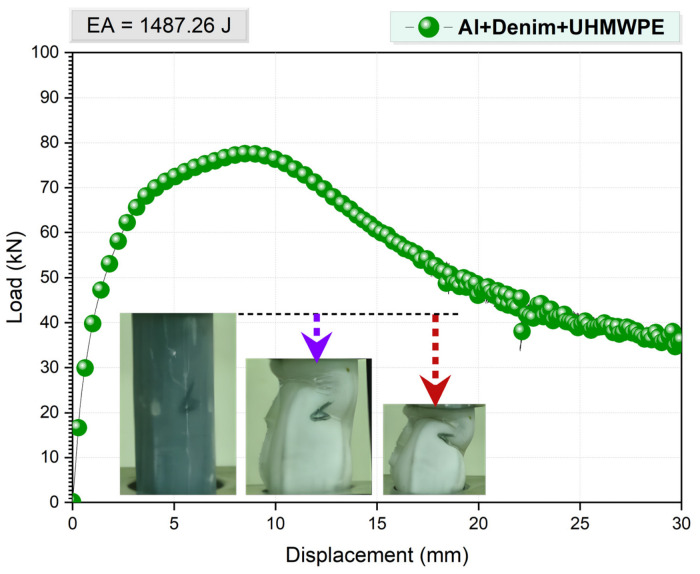
Length change and load-displacement graph of the Al+denim+UHMWPEFRP sample during the test.

Final sample, the Al+denim+UHMWPEFRP composite impact sample consisting of aluminum core material that was wrapped with denim and UHMWPE fibers showed results similar to the other combined samples. The peak force was 77.62 kN, which is higher than the values of the Al+denimFRP and Al+UHMWPEFRP samples. The calculated energy absorption value was 1487.26 J. This value is lower than the values obtained from the combined samples, Al+UHMWPEFRP and Al+denimFRP, which are 85.09 and 157.96 J, respectively. When the specific energy absorption value with a sample weight of 116.55 g was calculated, the value of 12.76 J/g was obtained. Compared with the Al+UHMWPEFRP and Al+denimFRP samples, this value was found to be lower by 1.3 and 2.76 J/g, respectively. The load-displacement graph for this sample is shown in Figure 8. No matrix or fiber fractures were observed during the test. Separation was observed only at the end of the contact point, the outer layer of which was wrapped with the UHMWPE fiber. As in the other combined samples, the aluminum exhibited a tendency to fold in the inner layer, and the denim and UHMWPE fibers wrapped on the outer layer tended to exhibit a reaction to this. These observations are in agreement with the load-displacement graphs seen in Figure 8 and during the test.

Energy absorption values, specific energy absorption values and sample weights of composite crash boxes are given in Table 3.

**Figure 9:**
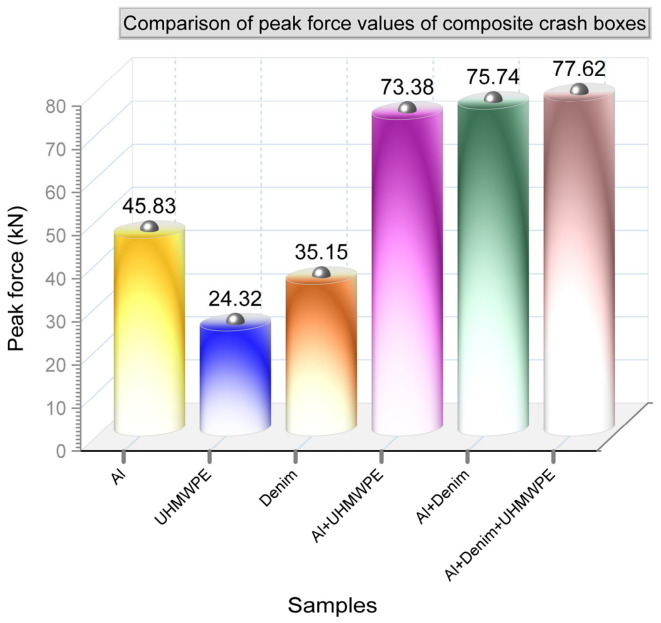
Comparison of peak force values of composite crash boxes.

The comparative graph of the peak force values of the samples is given in Figure 9.

As seen in Figure 9, the highest peak force value was achieved with the Al+denim+UHMWPEFRP sample. The Al sample, a metallic material, showed higher peak force values compared with the composite samples. One reason for the higher peak force results obtained from the combined samples is the aluminum material. With this combination, the peak force values reached high limits. Another noteworthy point here is that the DenimFRP sample exhibited a higher peak force value than the UHMWPEFRP sample. This can be interpreted as an indication that the denim fibers formed a good bond with the polymer matrix.

**Figure 10:**
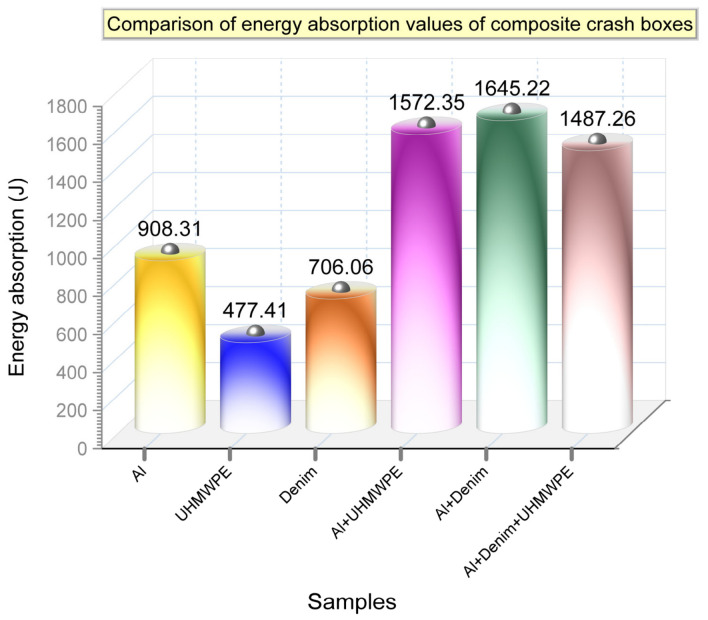
Comparison of energy absorption values of composite crash boxes.

Figure 10 shows graphical comparisons of energy absorption values of composite crash boxes.

As seen in Figure 10, the highest energy absorption value was obtained with the Al+denimFRP sample. Researchers have emphasized the effects of combination on composite materials [29,30,42,43]. The energy absorption values obtained through combined composites are higher than the total energy absorption values obtained individually for the two combined materials. This suggests that combining materials creates a good bond structure between the materials. In individually produced composite crash boxes, denimFRP has a higher energy absorption value than UHMWPEFRP, despite having a lower density. Denim fabrics are also known to have better properties compared with other cotton fabrics [44]. As a composite, denim formed a better bond and had a higher energy absorption value than ultra-high-molecular-weight polyethylene fiber. The composite crash box created by combining UHMWPE with aluminum also yielded energy absorption values close to Al+denimFRP.

**Figure 11:**
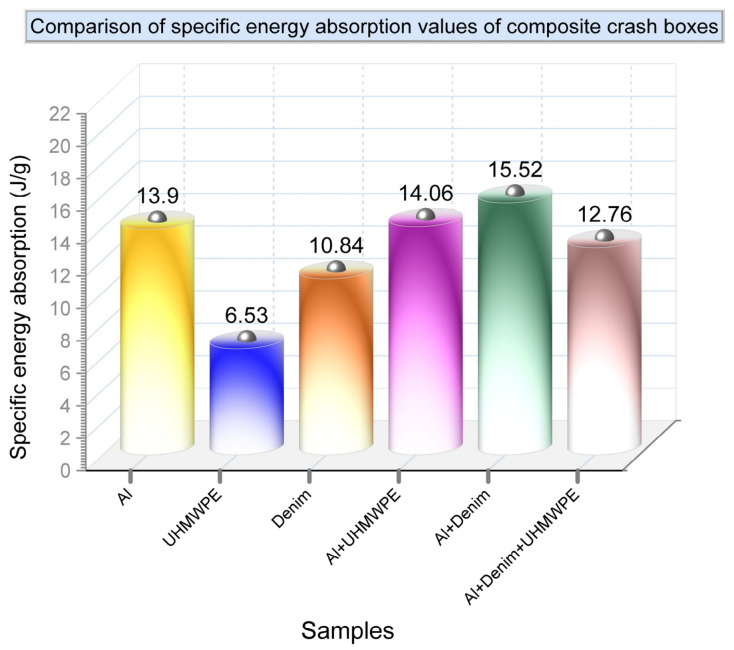
Comparison of specific energy absorption values of composite crash boxes.

A comparative graph of specific energy absorption values is given in Figure 11.

Specific energy absorption values generally yielded results similar to those of energy absorption. Specific energy absorption values are crucial for comparing different types of materials. Naturally, the highest values were obtained in combined samples, with aluminum, a metallic material, achieving higher values compared with other individual samples (Figure 11). As with the energy absorption value, the UHMWPEFRP sample also had the lowest value. The Al+denimFRP sample also achieved high values thanks to its excellent compatibility. Furthermore, the weft density used in denim fabric is higher than that of UHMWPE fabric, which also contributed to the better results of the Al+denimFRP sample. Aluminum, being a lightweight metal, is preferred in many engineering products due to its durability, especially when considering fuel efficiency in production, where aluminum easily finds its place in the automotive industry [45]. Denim fabrics are widely available on the market and have a significant place in industry. Mainly used as a textile product, denim fabrics are preferred over other fabrics due to their high strength. It is also important due to its sustainability [46]. Furthermore, the formation of a good interface bond with denim fabric and epoxy resin has resulted in high strength values. Combination with denim has also resulted in higher strength achievements than combination with UHMWPE. The fibers wrapped around the aluminum as a combination of materials tended to prevent the aluminum from protruding outwards around its axis during the semi-static compression test. It is believed that this preventive property led to an increase in the calculated values. UHMWPE exhibited similar behavior when used in combination with aluminum but showed lower energy absorption and specific energy absorption values than that of the combination with denim.

**Figure 12:**
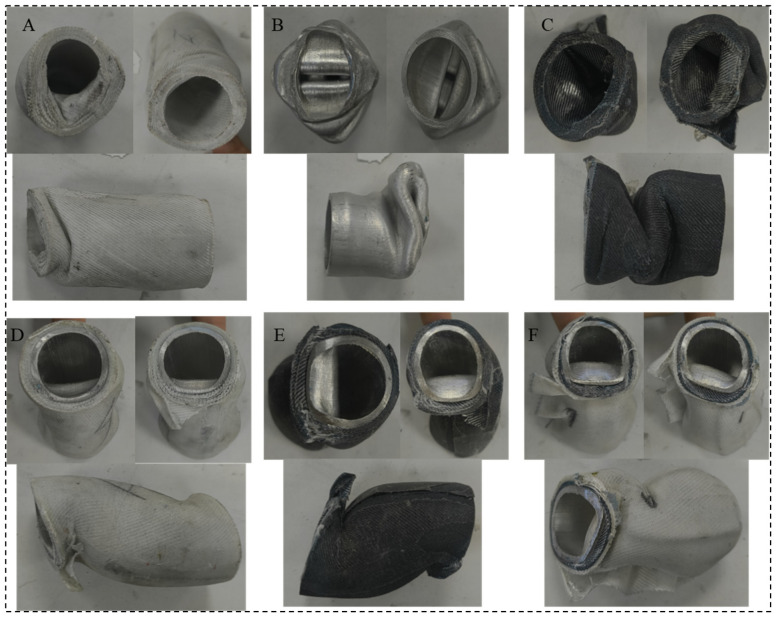
The final images of the samples after testing: (**A**) UHMWPEFRP, (**B**) Al, (**C**) DenimFRP, (**D**) Al+UHMWPEFRP, (**E**) Al+denimFRP, and (**F**) Al+denim+UHMWPEFRP.

Images obtained after the experiments on the samples are given in Figure 12.

## 4. Conclusions

In this study, three different mechanical properties of composite crash boxes were investigated. These were energy absorption, peak force, and specific energy absorption values. Different types of materials and their combinations were experimentally investigated using epoxy resin matrix composites. Aluminum, ultra-high-molecular-weight polyethylene, and denim were used as materials. As a result of the tests, the highest peak force was achieved with the composite crash box containing aluminum on the inside, denim fabric in the middle, and UHMWPE fabric on the outside. The best energy absorption values were achieved with the combined samples. Among the combined samples, Al+denimFRP achieved high energy absorption values. Another performance metric, specific energy absorption, was employed to evaluate the Al+denimFRP sample, which contained aluminum on the inside and exhibited the highest energy absorption value. The presence of aluminum as a metallic material in the interior and denim’s better epoxy absorption resulted in good combination results for these two. Furthermore, the fact that the weft density used in denim fabric is higher than that used in UHMWPE fabric also contributed to the better results of the Al+denimFRP sample. When the calculated and obtained values were compared during the tests, the lowest peak force, energy absorption, and specific energy absorption values were observed in the UHMWPEFRP sample with ultra-high-molecular-weight polyethylene fibers. Thus, it absorbed less epoxy compared with denim, contributing to these results. When the individual samples were compared, it was observed that Al stood out in terms of energy absorption, while the DenimFRP sample showed better results in terms of specific energy absorption. As for the combination, the combination of these two materials also paves the way for researchers to achieve high results. Consequently, the Al+denimFRP sample is recommended as the crash box-manufacturing composite since this combination has a positive effect on mechanical properties.

## Figures and Tables

**Table 1:** Some properties of the epoxy resin.

Properties of the Epoxy Resin	
Density (g/cm^3^)	1.13–1.17
Operating temperature before applying heat treatment (°C)	−60–+50
Operating temperature after applying heat treatment (°C)	−60–+80
Application temperature (°C)	+10–+50

**Table 2:** Specific properties of crash boxes.

Sample Name	Number of Denim Layers	Number of UHMWPE Layers
Al	-	-
UHMWPE**FRP**	-	9
Denim**FRP**	9	-
Al+UHMWPE**FRP**	-	6
Al+denim**FRP**	6	-
Al+denim+UHMWPE**FRP**	3	3

**Table 3:** Composite crash box peak force values, energy absorption values, specific energy absorption values and weights.

Crash Boxes	Peak Force (kN)	Energy Absorption (J)	Specific Energy Absorption (J/g)	Weight (g)
Al	45.83	908.31	13.90	65.34
UHMWPEFRP	24.32	477.41	6.53	73.10
DenimFRP	35.15	706.06	10.84	65.09
Al+UHMWPEFRP	73.38	1572.35	14.06	111.79
Al+denimFRP	75.74	1645.22	15.52	105.99
Al+denim+UHMWPEFRP	77.62	1487.26	12.76	116.55

## Data Availability

The original contributions presented in this study are included in the article. Further inquiries can be directed to the corresponding author(s).

## References

[1] Ciampaglia A., Patruno L., Ciardiello R. (2024). Design of a Lightweight Origami Composite Crash Box: Experimental and Numerical Study on the Absorbed Energy in Frontal Impacts. J. Compos. Sci..

[2] Alabtah F.G., Mahdi E., Khraisheh M. (2024). Energy absorption characteristics of a bio-inspired prepreg carbon fiber crash box under quasi-static axial compression. Compos. Part C Open Access.

[3] Ramalingam S.K., Selvan D.K., Prasath V.M.M., Balaji R.S., Loganathan P., Kumar A.N., Raghunathan V., Ayyappan V., Rangappa S.M., Siengchin S. (2026). 23-A crashworthiness study of composites for automobiles. Sustainable Composites for Automotive Engineering.

[4] Alkhatib F., Mahdi E., Dean A. (2020). Crushing response of CFRP and KFRP composite corrugated tubes to quasi-static slipping axial loading: Experimental investigation and numerical simulation. Compos. Struct..

[5] Allah M.M.A., El Aal M.I.A., El-baky M.A.A. (2024). Optimizing the crashworthy behaviors of hybrid composite structures through Taguchi approach. Polym. Compos..

[6] Allah M.M.A., Hegazy D.A., Alshahrani H., Sebaey T.A., El-baky M.A.A. (2023). Exploring the effect of nanoclay addition on energy absorption capability of laterally loaded glass/epoxy composite tubes. Polym. Compos..

[7] Boisse P., Akkerman R., Carlone P., Kärger L., Lomov S.V., Sherwood J.A. (2022). Advances in composite forming through 25 years of ESAFORM. Int. J. Mater. Form..

[8] Erkek B., Adin M.Ş., Kosedag E., Bronis M., Adin H. (2026). Effects of Artificial Hydrothermal Aging on Crush Boxes Made from Glass, Carbon and Aramid Fiber-Reinforced Hybrid Composites. Polymers.

[9] Kumar R.P., Muthukrishnan M., Sahayaraj A.F. (2023). Effect of hybridization on natural fiber reinforced polymer composite materials—A review. Polym. Compos..

[10] Erkek B., Kosedag E., Adin H. (2024). Hybridization effect on energy absorption capacity of composite crash boxes. Polym. Compos..

[11] Özen I., Gedikli H., Aslan M. (2023). Experimental and numerical investigation on energy absorbing characteristics of empty and cellular filled composite crash boxes. Eng. Struct..

[12] Kosedag E., Araz Z., Erkek B. (2025). Effect of Geometry and Fiber Type on Energy Absorption in Polymer Based Composite Crash Boxes: An Experimental Study. Polym. Compos..

[13] Bunsri P., Lophisarn S., Panpetch P., Jongpradist P., Kongwat S. (2024). Study on crushing behaviors of the crash box with truss-lattice and cellular structures. AIP Conf. Proc..

[14] Hussain N.N., Regalla S.P., Rao Y.V.D., Mohammed A.M. (2022). An experimental and numerical analysis on influence of triggering for composite automotive crash boxes under compressive impact loads. Int. J. Crashworthiness.

[15] Allah M.M.A., Hegazy D.A., Almuflih A.S., El-baky M.A.A. (2025). Experimental Optimization of Energy Absorption in Foam-Filled Square Multicell Composite Tubes Under Quasi-Static Loading. Polym. Compos..

[16] Saber A., Amer A.M., Shehata A.I., El-Gamal H.A., Abd_Elsalam A. (2025). Recent Developments in Additively Manufactured Crash Boxes: Geometric Design Innovations, Material Behavior, and Manufacturing Techniques. Appl. Sci..

[17] Tang G., She Z., Zhang Y., Li J., Feng R., Shu H. (2025). Design of a New Energy-Absorbing Box for Lightweight Electric Vehicles and Research on Vehicle Crashworthiness. World Electr. Veh. J..

[18] Bai C., Zhou T., Ma Q., Gan X. (2022). Multi-objective crashworthiness optimization of CFRP/Al hybrid tube under transverse loading. J. Reinf. Plast. Compos..

[19] Erkek B., Kosedag E., Adin H. (2025). The impact of graphene filler on the energy absorption of hybrid composite crash boxes. Int. J. Mech. Mater. Des..

[20] Zhu G., Zhao X., Shi P., Yu Q. (2019). Crashworthiness Analysis and Design of Metal/CFRP Hybrid Structures Under Lateral Loading. IEEE Access.

[21] El Aal M.I.A., Allah M.M.A., El-baky M.A.A. (2023). Carbon-glass reinforced epoxy hybrid composites for crashworthy structural applications. Polym. Compos..

[22] Luo H., Zhang D., He Z., Li X., Li Z. (2021). Experimental investigation of the quasi-static and dynamic axial crushing behavior of carbon/glass epoxy hybrid composite tubes. Mater. Today Commun..

[23] Allah M.M.A., Hegazy D.A., Alshahrani H., Sebaey T.A., El-baky M.A.A. (2024). Crush Analysis of Hybrid Glass/Nano-Silica/Epoxy Composite Cylinders Under Lateral Loading Conditions. Fibers Polym..

[24] Bedi S.S., Mallesha V., Mahesh V., Ponnusami S.A. (2024). Investigation of low-percentage graphene reinforcement on the mechanical behaviour of additively manufactured polyethylene terephthalate glycol composites. J. Thermoplast. Compos. Mater..

[25] El-Ghazaly A., Anis G., Salem H.G. (2017). Effect of graphene addition on the mechanical and tribological behavior of nanostructured AA2124 self-lubricating metal matrix composite. Compos. Part Appl. Sci. Manuf..

[26] Li Y., Feng Z., Huang L., Essa K., Bilotti E., Zhang H., Peijs T., Hao L. (2019). Additive manufacturing high performance graphene-based composites: A review. Compos. Part Appl. Sci. Manuf..

[27] Kösedağ E. (2023). Investigation of the Effect of Filling Ratio on Mechanical Properties of Pumice Filled Epoxy-Based Composites. Gazi Üniversitesi Fen Bilim. Derg. Part C. Tasar. ve Teknol..

[28] El-baky M.A.A., Allah M.M.A., Kamel M., Abdel-Aziem W. (2023). Fabrication of Glass/Jute Hybrid Composite over Wrapped Aluminum Cylinders: An Advanced Material for Automotive Applications. Fibers Polym..

[29] Zhang C., Sun Y., Bui T.Q., Curiel-Sosa J.L. (2024). Crushing performance and energy absorption characteristics of aluminum/CFRP hybrid thin-walled tubes: Experimental and numerical investigations. Compos. Commun..

[30] Hwang S.-F., Wu C.-Y., Liu H.-K. (2021). Crashworthiness of Aluminum-Composite Hybrid Tubes. Appl. Compos. Mater..

[31] Kalayci E., Avinç O.O., Yavaş A. (2016). YÜKSEK PERFORMANSLI POLİETİLEN (HPPE) LİFLERİ. Marmara Fen Bilim. Derg..

[32] Anwer A.A., Dong T., Naguib H.E. (2020). Fiber tortuosity and its effects on shock transfer characteristics of Ultra High Molecular Weight Polyethylene (UHMWPE) fibers embedded in a polyurethane composite structure. Compos. Sci. Technol..

[33] Ren Y., Huang W., Gong J., Zeng J., Zhong G., Li Z., Fu Q., Gao X. (2025). HDPE/UHMWPE composite pipe: A systematic study from preparation process to performance enhancement. Polymer.

[34] Khatiwada S., Armada C.A., Barrera E.V. (2013). Hypervelocity Impact Experiments on Epoxy/Ultra-high Molecular Weight Polyethylene Fiber Composites Reinforced with Single-walled Carbon Nanotubes. Procedia Eng..

[35] Jian L., Gaofeng X. (2024). The effect of CNT grafting on the mechanical properties of the UHMWPE fiber/HDPE composite. Surf. Interface Anal..

[36] Koç E., Ayyıldız Ç. (2005). Denim Kumaş Üretim Esaslari, Dünya Ve Turkiye’deki Ticaret Durumu. Tekst. ve Mühendis.

[37] Hassani P., Soltani P., Ghane M., Zarrebini M. (2021). Porous resin-bonded recycled denim composite as an efficient sound-absorbing material. Appl. Acoust..

[38] Webo W., Khoathane M.C., Mhike W., Mohlamonyane R.S. (2025). Enhancing sustainability: Composite materials from recycled HDPE and denim fillers. Polym. Eng. Sci..

[39] Aman, Tonk D., Shokeen K., Singh D. (2022). Development of fire retarding composite board for fire compartmentation application using waste denim: A review. Mater. Today Proc..

[40] Öztemur J., Sezgin H., Enis I.Y. (2021). Design of an Impact Absorbing Composite Panel from Denim Wastes and Acrylated Epoxidized Soybean Oil based Epoxy Resins. Tekst. ve Konfeksiyon.

[41] Temmink R., Baghaei B., Skrifvars M. (2018). Development of biocomposites from denim waste and thermoset bio-resins for structural applications. Compos. Part A Appl. Sci. Manuf..

[42] Girimurugan R., Pugazhenthi R., Suresh T., Maheskumar P., Vairavel M. (2021). Prediction of mechanical properties of hybrid aluminium composites. Mater. Today Proc..

[43] Bhong M., Khan T.K., Devade K., Krishna B.V., Sura S., Eftikhaar H., Thethi H.P., Gupta N. (2023). Review of composite materials and applications. Mater. Today Proc..

[44] Natarajan G., Rajan T.P. (2024). Mechanical Properties of Cotton and High-Performance Fiber Blended Denim Fabrics for Motorcycle Protective Clothing. J. Test. Eval..

[45] Cole G., Sherman A. (1995). Light weight materials for automotive applications. Mater. Charact..

[46] Sarı B., Zarifi F., Alhasan M., Güney H., Türkeş S., Sırlıbaş S., Yiğit D.C., Kılınççeker G., Şahin B., Keskinkan O. (2023). Determining the Contributions in a Denim Fabric Production for Sustainable Development Goals: Life Cycle Assessment and Material Input Approaches. Sustainability.

